# Testicular–Epididymal Dissociation: Vas and Vessels May “Lead up the Garden Path”

**DOI:** 10.1055/s-0039-1688485

**Published:** 2019-12-13

**Authors:** Eleni Papageorgiou, Alberto Mantovani, Elena Monti, Caroline Brain, Naima Smeulders, Abraham Cherian

**Affiliations:** 1 Department of Paediatric Urology, Great Ormond Street Hospital For Children NHS Foundation Trust, London, United Kingdom of Great Britain and Northern Ireland; 2 Department of Paediatric Endocrinology, Great Ormond Street Hospital For Children NHS Foundation Trust, London, London, United Kingdom of Great Britain and Northern Ireland

**Keywords:** laparoscopy, intra-abdominal testis, embryological descent

## Abstract

The vas deferens and spermatic vessels entering the inguinal canal through the internal inguinal ring is thought to exclude an intra-abdominal testis. We present a case of high bilateral intra-abdominal testes on a 46,XY boy despite the vas deferens and good-sized vessels passing through the deep rings.

Data were collected from clinical records, radiology (ultrasound, magnetic resonance imaging [MRI]), and endocrine blood tests.

This case underlines the importance of following the pathway of embryological descent of the testis cranially as well as caudally during diagnostic laparoscopy, to avoid missing this rare anatomical variant.

## Introduction

Laparoscopic findings in case of impalpable undescended testes have been well described in the literature. When both the vas and vessels enter the internal inguinal ring, an exploration is generally indicated. However, in the absence of blind-ending vessels in the proximity of the internal inguinal ring, laparoscopic exploration must continue in a cranial direction to locate a possible high intra-abdominal testis.

We present a unique case in which vas and vessels were entering the internal inguinal ring but the testes were found to be intra-abdominal. The diagnosis turned out to be a long bilateral testicular–epididymal dissociation (TED).

## Case Report

A newborn, term baby had been referred to the local urology department for severe penoscrotal hypospadias and bilateral impalpable testes. The genetic and endocrine investigations confirmed a 46,XY karyotype as well as a normal response to Hcg (human chorionic gonadotropin) stimulation. Diagnostic laparoscopy at 1 year of age demonstrated the vas deferens and vessels entering the internal inguinal rings bilaterally. Groin exploration revealed what was considered to be atrophic testicular nubbins, which were left in situ at parental request. Hypospadias correction was performed separately. Throughout childhood, the endocrine status revealed normal hormonal levels ( Table 1 ).

**Table 1:** Hcg test responses and gonadotropins levels at 1.5 and 3 years of age

	Testosterone (nmol/L)	LH (IU/L)	FSH (IU/L)
1.5 y	0.2–6.8	0.1 (<0.7)	2.4 (0–0.7)
3 y	0.3–4.2	<0.5 (<0.7)	0.8 (0–0.7)

Abbreviations: FSH, follicle-stimulating hormone; Hcg, human chorionic gonadotropin; LH, luteinizing hormone.

Spontaneous puberty and inability to locate palpable gonads triggered further investigations. Endocrine profiling at this point showed normal testosterone levels sustained by high gonadotropins. The anti-Müllerian hormone (AMH) and Inhibin-B levels were at the lower end of the reference range, suggesting functioning testicular tissue ( Tables 2 and 3 ).

**Table 2:** GnRH test at age 9.5 years and basal gonadotropins throughout puberty (9.5–17.5 years)

	LH (IU/L)	FSH (IU/L)	Testosterone (nmol/L)
9.5 y GnRH at 20'-60'	<0.2 6.9–4.7	3.1 9.1–13	< 0.69
10 y (normal ranges)	0.8 (0.3–1.4)	5.8 (0.5–6.4)	< 0.69
11.7 y (normal ranges)	7.9 (0.3–1.4)	21.2 (0.5–6.4)	7.97
13.2 y (normal ranges)	10.2 (0.4–0.6)	24.4 (0.7–6.9)	15.6
15.7 y (normal ranges)	25 (1.7–8.6)	35.9 (1.5–12.4)	16.8 (7.6–31.4)
17.5 y (normal ranges)	30.8 (1.7–8.6)	46.9 (1.5–12.4)	14.3 (7.6–31.4)

Abbreviations: FSH, follicle-stimulating hormone; GnRH, gonadotropin-releasing hormone; Hcg, human chorionic gonadotropin; LH, luteinizing hormone.

**Table 3:** Three-week Hcg test at age 9.5 years

Time	0	3 wk
DHEA-S (μmol/L) (0.4–2)	0.866	1.66
Androstenedione (nmol/L)	<1.05	3.5
Testosterone (nmol/L)	<0.69	15.5
DHT (nmol/L)	<0.1	0.27
Inhibin B (pg/mL)	74.2 (50–310)	
AMH (pmol/L)	116.2 (134–184)	

Abbreviations: AMH, anti-Müllerian hormone; DHEA-S, dehydroepiandrosterone sulfate; DHT, dihydrotestosterone; Hcg, human chorionic gonadotropin.

**Fig. 1:**
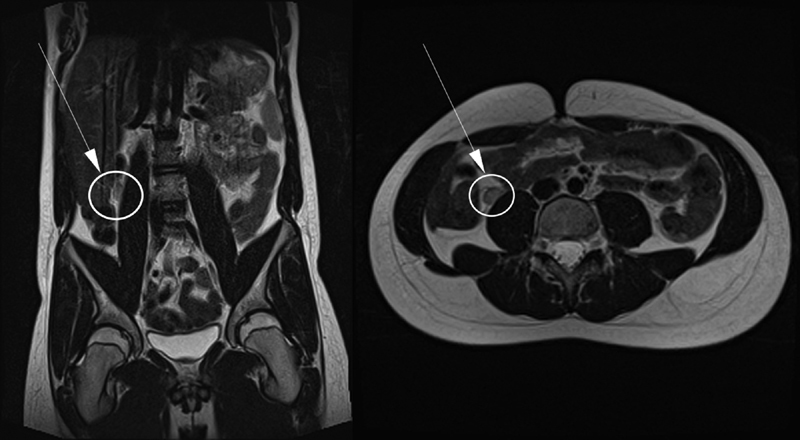
Magnetic resonance imaging (MRI).

**Fig. 2:**
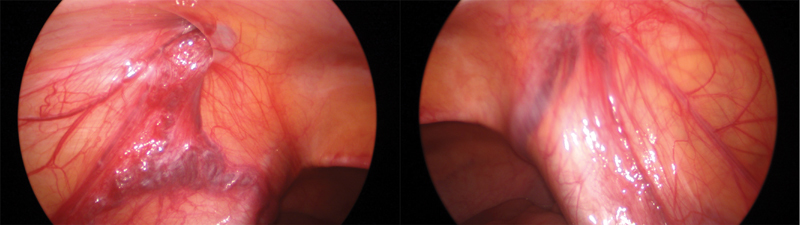
Laparoscopy: vas and vessels entering deep inguinal rings.

**Fig. 3:**
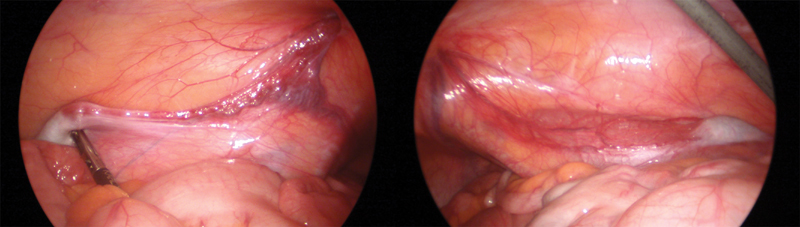
Laparoscopy: testis on the psoas muscle abutting the lower pole of the kidney.

The discrepancy between good hormonal response at a repeat Hcg test and the ongoing impalpable nature of the gonads led to the question of possibly missed intra-abdominal testes. Ultrasound scan failed to detect any gonads; however, subsequent abdominal magnetic resonance imaging (MRI) suggested intra-abdominal testes close to the lower pole of the respective kidneys ( Fig. 1 ). Family and patient consented to a diagnostic laparoscopy but no intervention: this confirmed vas and vessels entering the deep inguinal rings ( Fig. 2 ); however, further mobilization of colon medially allowed the vessels to be traced from the internal ring cranially to finally reveal the testis on the psoas muscle abutting the lower pole of each kidney ( Fig. 3 ). The left testis has been considered suitable for a Fowler–Stephen two-stage laparoscopic orchidopexy, whereas the right testis showed a poor vascular pedicle, and orchidectomy has been recommended.

## Discussion

Testis and epididymis follow differing embryological pathways: the former originates from the urogenital crest, whereas the latter derives from the mesonephric duct. The SRY (Sex-determining Region of Y-chromosome) gene expression guides the undifferentiated sex cords to transform into seminiferous cords first and *rete testes* later. 1 At 6 months of gestation, *rete testes* and the mesonephric tubules share a common lumen. 2 The epididymis always precedes the testicle in the descent through the groin into the scrotum and is constantly anatomically distal to the gonad. 3 Fusion defects between epididymis and testicle can occur at different levels of the descent pathway and in different portions of the epididymis. Turek et al in 1994 defined the normal epididymal anatomy: the “looped” body epididymis connected with the testis by head and tail (type-1) is the most common configuration (83.9%) followed by the complete attachment of the epididymis to the testis (12.5%). Head or tail attachment only and complete nonfusion were the rarest varieties. 4 The incidence of fusion defects is higher in an undescended testicle. 5 Windholz classified fusion abnormalities with undescended testis into four categories: (1) absent testis with epididymis/vas deferens within the scrotum, (2) undescended testis with epididymis/vas deferens within the scrotum, (3) undescended testis and epididymis with only the vas deferens within the scrotum, and (4) both testis and epididymis descended within the scrotum but nonfused. 6

The classifications proposed by Turek et al and Windholz focused on features of aberrant fusion but lacked a qualitative description of the epididymis in its different portions in case of fusion defects. The epididymis can, in fact, be also maldeveloped. Simon included in their classification qualitative aspects of the fusion defect. They categorized cases of head attachment achieved by elongated efferent ducts (type b), as well as direct testis-vas deferens fusion with absent epididymis (type c), absent epididymal body (type d), and absent epididymal head and body (type e). 7 8 Variations among classifications account for many possible anatomical variants in terms of both fusion defect and segmental maldevelopment. TEDs can subsequently be represented by a pure fusion defect, a segmental structural maldevelopment, or their association. Our case is probably a fusion defect of intra-abdominal high testis characterized by absent epididymal head and body (type-e, Simon's classification), replaced by fibrotic structures, with descended epididymal tail and vas deferens, where the tail was probably originally misdiagnosed as testicular remnant bilaterally. 9 However, the lack of the original laparoscopic appearances makes accommodation of our case into a particular classification debatable. In retrospect, an MRI scan after the first surgery at the first year of age, in light of the positive Hcg test, as well as the AMH and inhibin results, would have been helpful. However, we are describing historical data, over which we could have no control.

Laparoscopy is considered the gold standard for the assessment of intra-abdominal testes. Once both vas and vessels are seen entering the internal inguinal ring, an inguinal exploration is generally advised. On the contrary, if both vas and vessels are blind ending, an inguinal exploration should be theoretically unnecessary. 10 Snodgrass et al demonstrated scrotal nubbins associated with blind ending vas and vessels. 11 When a normal vas without vessels in its proximity is seen entering the internal inguinal ring, a strong recommendation is to look for high intra-abdominal testes. In our case, the vessels entering the internal inguinal ring bilaterally could be the epididymal vessels. These arteries are usually branches of the internal spermatic artery: three epididymal arteries usually arise from the epididymis to serve the head, body, and tail independently. The epididymal venous drainage is through the pampiniform plexus, and varicosity of the vessels on the left side of this case could represent that contribution. 12

As the appearance of the nubbins was initially strongly indicative of gonadal remnants, we have also retrospectively postulated the polyorchidism variant. This is more common on the left side and can be associated with cryptorchidism. 13 Leung described four types of polyorchidism based on the different relations that the supernumerary testis can have with the epididymis and vas. 14 In type 3 of Leung's classification, in fact, the supernumerary, proximal testis has its own epididymis but shares a common vas with the distal testis. However, the absence of two independent testicular vascular systems excludes this hypothesis. Polyorchidism is also difficult to correlate with the findings on the right side, where the vas deferens remnant is clearly following a normal pathway.

A two-stage Fowler–Stephen procedure has been offered for the left testicle as TEDs do not correlate with significant histological gonadal abnormalities; therefore, orchidopexy remains an option if the testicle is eligible for it. 15

## Conclusions

The incidence of testicular–epididymal fusion defects is high for undescended intra-abdominal testes, and, at laparoscopy, vessels deriving from an aberrant fusion attempt might be seen. Those vessels, if examined at the level of the internal inguinal ring only, can be indistinguishable from normal retroperitoneal spermatic artery and veins. If only a nubbin is found at exploration and the preoperative Hcg test is positive, suggesting functioning testicular tissue, a careful search along the pathway of testicular descent, with colonic mobilization, looking for high intra-abdominal gonads, is therefore strongly recommended to avoid missing the anatomical variant presented.

## Notes

New Insights and the Importance for the Pediatric Surgeon A careful search along the pathway of testicular descent is recommended to avoid missing the anatomical variant of testicular–epididymal fusion defects.
