# Supplementary material for: Testicular–Epididymal Dissociation: Vas and Vessels May “Lead up the Garden Path”
Source: European J Pediatr Surg Rep. 2019 Dec 13;7(1):e96–9. doi: 10.1055/s-0039-1688485 (PMC6923713; doi:10.1055/s-0039-1688485)
Supplement: Supplementary file 1 — Supplementary Material [file 10-1055-s-0039-1688485-s180410cr.pdf]

Supplementary Table S1 Leung's classification of polyorchidism

Type	Anatomical feature
I	Supernumerary testis lacking epididymis and vas deferens
II	Supernumerary testis shares the epididymis and vas of the other testis
III	Supernumerary testis has its own epididymis but shares the vas deferens with the other testis
IV	Complete duplication of testes, epididymis, and vas deferens

Note: adapted from Leung AK. Polyorchidism. Am Fam Physician 1988;35:15–156.
